# The Vulnerability of Threatened Species: Adaptive Capability and Adaptation Opportunity

**DOI:** 10.3390/biology2030872

**Published:** 2013-07-01

**Authors:** Pam Berry, Yuko Ogawa-Onishi, Andrew McVey

**Affiliations:** 1 Environmental Change Institute, University of Oxford, OUCE, South Parks Road, Oxford, OX1 3QY, UK; 2 Center for Social and Environmental Systems Research, National Institute for Environmental Studies, 16-2 Onogawa, Tsukuba, Ibaraki 305-8506, Japan; E-Mail: onishi.yuko@nies.go.jp; 3 WWF-UK, Panda House, Weyside Park, Godalming, Surrey GU7 1XR, UK; E-Mail: AMcVey@wwf.org.uk

**Keywords:** climate change, adaptation capacity, vulnerability, adaptation capability, adaptation opportunity, conservation, mammals, priority species, marine

## Abstract

Global targets to halt the loss of biodiversity have not been met, and there is now an additional Aichi target for preventing the extinction of known threatened species and improving their conservation status. Climate change increasingly needs to be factored in to these, and thus there is a need to identify the extent to which it could increase species vulnerability. This paper uses the exposure, sensitivity, and adaptive capacity framework to assess the vulnerability of a selection of WWF global priority large mammals and marine species to climate change. However, it divides adaptive capacity into adaptive capability and adaptation opportunity, in order to identify whether adaptation is more constrained by the biology of the species or by its environmental setting. Lack of evidence makes it difficult to apply the framework consistently across the species, but it was found that, particularly for the terrestrial mammals, adaptation opportunities seems to be the greater constraint. This framework and analysis could be used by conservationists and those wishing to enhance the resilience of species to climate change.

## 1. Introduction

The review of the status and trends of global biodiversity showed that the target “to achieve by 2010 a significant reduction of the current rate of biodiversity loss at the global, regional and national level as a contribution to poverty alleviation and to the benefit of all life on Earth” has not been met [1]. This target was also incorporated into the Millennium Development Goals, as biodiversity underpins much of human health and well-being [2]. Biodiversity, therefore, is an integral part of human life and its conservation in the face of many pressures is important. There are five main pressures on biodiversity: climate change, habitat loss and degradation, excessive nutrient load and other forms of pollution, over-exploitation, and unsustainable use and invasive alien species [3]; it is expected that these will continue to exert pressure in most areas during the 21st century [4]. For many species, climate change is not yet the most significant direct threat in the short-term [5,6], but for those that are at risk from other pressures, climate change can be an additional factor that leads to their decline. In addition, as climate change continues, greater impacts are projected, while ecosystem and species responses may be lagged [7]. Thus there is a need to identify the extent to which climate change could increase threatened species vulnerability and lead to a failure to meet Aichi Target 12 of “preventing the extinction of known threatened species and their conservation status...has been improved and sustained” [8].

Species vulnerability to climate change has been assessed in various ways; some use these three components (e.g., [9,10]) while, in the absence of specific criteria, the IUCN focused on using species traits to cover aspects of the possible sensitivity and adaptive capacity of the species [11]. A more complex framework used by the National Wildlife Federation [12], characterizes both the current (non-climate) stressors of threatened and endangered species in the U.S. and the potential effects of climate change on species vulnerability, before combining them into an overall evaluation of potential future vulnerability.

One reason for assessing species vulnerability is to guide and, in some cases, prioritize species-level conservation efforts. A range of species and habitat related adaptation options have been suggested (e.g., [13,14]) to reduce vulnerability through enhancing adaptive capacity. This paper, while adopting the IPCC approach to vulnerability, argues that for adaptive capacity it is important to distinguish between a species adaptive capability and its adaptation opportunity. The former is a function of the species ability to respond autonomously to climate change through distributional, phenotypic and genotypic and/or evolutionary changes. The latter is a function of opportunities for these changes to be expressed in the context of other (human) drivers of change, which are often not explicitly assessed. It is seen most clearly in the availability of habitat to enable species to migrate or stresses that reduce population size, such that they are more susceptible to other stresses and few individuals migrate. The usefulness of the distinction and its application to identifying conservation action was tested by assessing the vulnerability of selected WWF Global Priority terrestrial mammals and marine species. It was also used to assess how conservation can address this vulnerability through enhancing adaptive capacity.

## 2. Method

In order to assess species vulnerability we followed the Intergovernmental Panel on Climate Change (IPCC) [15,16] definitions and did not use the terminology defined by a recent IPCC report [17] on managing the risks of extreme events and disasters to advance climate change adaptation (SREX), as the latter focuses on risk management by humans and the definitions are not directly transferable (or applicable) to species vulnerability assessments. The vulnerability of a species to climate change, therefore, can be considered to depend on the species exposure (the nature and degree to which a system is exposed to significant climatic variations (Glossary, IPCC, 2001)); its sensitivity (the degree to which a system is affected, either adversely or beneficially, by climate variability or change (Glossary, IPCC, 2007)); and its adaptive capacity (the ability of a system to adjust to *climate change* (including *climate variability* and extremes) to moderate potential damages, to take advantage of opportunities, or to cope with the consequences (Glossary, IPCC, 2007)).

### 2.1. Species Selection

WWF global priority species were used as they include some of the most threatened and ecologically, culturally and economically important species [18]. Terrestrial large mammals and a range of marine species were chosen to provide possible contrasts in climate vulnerability and to test the framework in two contrasting environments.

### 2.2. Vulnerability Assessment

In order to identify species vulnerability and what conservation action is most needed, their exposure, sensitivity, adaptive capability and adaptation opportunity were assessed, based on a literature review and climate projections, with expert knowledge to fill gaps where possible (Table 1).

**Table 1:** The components of the vulnerability assessment for threatened species.

	Exposure	Sensitivity	Adaptive capability	Adaptation opportunity
Concepts	Magnitude of changes in climate experienced by species	Susceptibility of species to climate change (degree of species responses per unit of climate variable change)	Ability to occupy areas that become suitable under climate change	Physical factors that enable or limit species adaptive capability
			Ability to persist when climate becomes unsuitable	
Indicators	Projected changes in temperature across the species range	Physiological tolerance limits	Dispersal rate/ability	Projected changes in habitats or climatically suitable areas
	Projected changes in precipitation across the species range	Association with sensitive/restricted habitats	Evolutionary potential (e.g., genetic diversity, generation times, population size)	Landscape connectivity/barriers to migration
	(Changes in extreme events) *	Specialist feeder	Ecological plasticity	Availability of food sources
	(Sea level rise) *	Degree of phenology changes		Land use change pressures
		Sensitivity to extremes/dependence on environmental triggers		Presence/improvement of protected areas
		Life history traits		Other pressures (e.g., poaching, hunting, trade)
		Population dynamics		

* Factors that were not used in this assessment of exposure.

The peer-reviewed literature was systematically searched using Web of Knowledge, with species name, climate change and other vulnerability-related parameters as search terms (Table 2), and by following up citations and using literature known to the authors, supplemented by grey literature and documents provided by WWF. It included species-relevant papers up until March 2013.

**Table 2:** Search terms used in conjunction with the species’ name for the literature review.

Vulnerability parameter	Search term
Pressure	Climate change
	Global warming
	Environmental change
Exposure	Temperature
	Rainfall
	Drought
Sensitivity/Adaptive capacity	Range shift
	Distribution shift
	Habitat shift

Species exposure was established using temperature and precipitation data from the global circulation models (GCM) in IPCC Fourth Assessment Report [19], downscaled to a 2.5 minute resolution [20]. These two parameters are the most reliable outputs from GCMs and also significant for species’ distributions and functioning. Data from HadCM3 GCM, one of the more widely used GCMs, was used for this analysis. In order to capture some of the range of uncertainty in future projections, two emissions scenarios were used: a higher emission scenario (SRES A2) and lower emission scenario (SRES B1). The data were developed using a change-factor method [21], with the interpolated climate surfaces of Worldclim [22] as the 20th century climate baseline. Thus, the climate changes were calculated for the species range by subtracting the current climates from the climate projections for 2050 (2041–2060). For marine species, changes in sea surface temperatures (SST) were calculated for the same period based on HadCM3 under SRES A2 [23]. Species’ range data were obtained from the IUCN Red List of Threatened Species [24] and the State of the World’s Sea Turtles [25,26,27] for turtles.

Exposure was categorized using arbitrary thresholds (Table 3), with temperature categorized using increases in mean annual temperature, with below 2 °C being seen as a target for climate change negotiations and policy, and beyond which many more ecosystems could be negatively affected [28], while 4 °C has been proposed as a “tipping point” for the climate system, although this can vary according to the component under consideration [29]. It should be noted that where the current precipitation totals are low, e.g., African rhinoceros and snow leopard, then the percentage changes can be very high, even though the absolute changes are relatively small. In addition, seasonal variations and extremes, which can be more significant than mean changes, are not taken into account. The assessment for the marine species also requires caution, as many are wide-ranging, and it is difficult to establish in which parts of their range they would be most sensitive to climate change and many of them utilize different depths. Details of changes in climate variables in the species ranges are provided in supplementary materials (Table 4, Table 5).

**Table 3:** Climate change exposure categories.

Increase in mean annual temperature (°C)	Exposure category
Less than 2 °C	Low
2–4 °C	Medium
More than 4 °C	High
Change in annual precipitation (%)	
Between −5 to +5	Low
Either >5 or <10 decrease or increase	Medium
More than 10 decrease or increase	High

**Table 4:** Projected changes in temperatures and precipitation in species’ distribution range (terrestrial species).

	SRES A2						SRES B1					
Species	Temperature increase (°C)			Precipitation change (%)			Temperature increase (°C)			Precipitation change (%)		
	Min	Max	Mean	Min	Max	Mean	Min	Max	Mean	Min	Max	Mean
African Elephant	0.7	4.2	2.3	−93	28	−2	−0.1	3.0	1.8	−91	17	−5
White Rhinocero	0.6	3.1	2.4	−100	100	−14	0.3	2.5	1.8	−100	82	−14
Black Rhinocero	0.6	3.1	2.3	−100	100	−14	−0.1	2.5	1.8	−100	82	−14
Western Gorilla	1.1	2.3	1.9	−51	8	−3	0.7	1.9	1.5	−39	3	−6
Eastern Gorilla	2.0	2.3	2.1	−2	1	−1	1.5	1.8	1.6	−4	−1	−3
Chimpanzee	1.0	2.5	2.1	−58	17	2	0.6	2.2	1.6	−48	9	−2
Bonobo	2.1	2.4	2.3	−3	10	6	1.6	1.9	1.8	−10	4	0
Bornean Orangutan	1.3	2.1	1.7	−10	9	4	0.9	1.6	1.2	−12	7	2
Sumatran Orangutan	1.3	1.8	1.5	−12	1	−6	1.0	1.4	1.2	−16	5	−4
Asian Elephant	1.3	2.5	1.9	−20	29	2	1.0	2.1	1.5	−23	20	0
Indian Rhinocero	1.8	2.1	1.9	2	10	7	1.2	1.8	1.4	−1	12	5
Javan Rhinocero	1.5	2.0	1.5	−8	-5	−7	1.0	1.6	1.1	−6	−5	−6
Sumatran Rhinocero	1.4	2.0	1.7	−14	11	−6	1.0	1.6	1.3	−18	14	−5
Snow Leopard	1.7	3.0	2.3	−71	221	21	1.0	2.8	1.9	−36	229	20
Clouded Leopard	1.4	2.3	2.0	−20	37	8	1.2	2.2	1.7	−23	42	2
Sundaland Clouded Leopard	1.3	2.1	1.7	−13	12	2	0.9	1.6	1.3	−16	17	2
Tiger	1.3	3.3	2.1	−20	32	5	1.0	3.1	1.7	−24	35	3
Giant Panda	1.9	2.3	2.2	2	12	6	1.5	2.2	1.9	1	5	3

**Table 5:** Projected changes in sea surface temperatures (SST) in species’ distribution range (marine species).

	SRES A2		
Species	SST increase (°C)		
	Min	Max	Mean
Loggerhead turtles	−1.2	4.9	1.3
Hawksbill turtles	−1.0	2.9	1.3
Green turtle	−1.2	3.5	1.2
Leatherback turtle	−3.0	4.9	1.2
Olive ridley turtle	−1.0	3.8	1.3
Kemp’s turtle	−1.2	2.7	0.8
Blue whale	−3.0	4.9	1.1
North Atlantic right whale	−3.0	2.7	0.6
Bowhead whale	−3.0	3.1	0.7
Fin whale	−3.0	4.9	1.1
Gray whale	0.2	3.8	1.1
Sei whale	−3.0	4.9	1.1

Sensitivity was taken as responsiveness to changes in climate variables. A robust classification of species’ sensitivity, which can be compared across species, is not possible, as there is insufficient data on the response of species to the climate parameters. The assessment, therefore, was based on expert judgment of the evidence, including underlying species traits that affect the species responses and the number of climate change parameters to which the species is sensitive.

Adaptive capability was assessed by recording responses to current climate change, which could be considered part of autonomous adaptation. As for sensitivity, there is a paucity of data and a crude classification was used. Low adaptive capability was assumed when there was no evidence of any current or potential adaptation response or there was possible evidence of a difficulty in adapting, medium adaptive capability where there is some evidence of current or potential adaptation and high adaptive capability s where there was evidence of several forms of actual or potential adaptation.

Adaptation opportunity was assessed, as for adaptive capability, in terms of the amount of evidence of the effects of other pressures for change, which might impact directly or indirectly and positively or negatively on species being able to express their adaptive capability (Table 1). Adaptation opportunity is very difficult to assess for marine species, but some of the key pressures affecting terrestrial species, such as habitat loss and fragmentation and barriers to movement are not so evidently present for marine species, although various forms of harvesting may be important and parallel poaching and hunting. In the absence of specific evidence, such as overexploitation affecting population numbers, they were all assumed to have at least medium adaptation opportunity.

## 3. Results

### 3.1. Exposure

Under the SRES B1 scenario, no species were projected to experience mean temperature increases of more than 2 °C, thus all the species were classified as low exposure (Table 6), although the maximum projected temperature could exceed this value by more than 0.5 °C for African elephants and rhinoceros. Only the latter were classified as having a high exposure to precipitation changes under this scenario, but, as flagged earlier, this is a function of the relatively low current rainfall totals and the coarse resolution of the original climate model. Many other species also showed a wide range in projected precipitation change for the same reason, but this could also be a function of the greater uncertainty in modeling precipitation.

**Table 6:** Vulnerability of species to climate change.

Species	Exposure A2		Exposure B1		Sensitivity	Adap. Cap.	Adap. Opp.	Other pressures
	Temp	Rain fall	Temp	Rain fall				
Terrestrial mammals								
African elephant	M	L	L	L	H	M		Poaching, habitat degradation.
Asian elephant	L	L	L	L	n.e.	M	L	Shifting cultivation, encroachment, habitat fragmentation, poaching, mining, forest fire, water scarcity in dry season, increase in human populations, man-elephant conflict and mortality due to diseases.
White rhinoceros	M	H	L	H	n.e.	L	L	Poaching
Black rhinoceros	M	H	L	H	n.e.	n.e.	L	Food scarcity in drought years
Indian rhinoceros	L	M	L	L	n.e.	M	L	Poaching, habitat loss and degradation
Javan rhinoceros	L	M	L	M	n.e.	L	L	Poaching, forest clearance
Sumatran rhinoceros	L	M	L	L	n.e.	L	L	Poaching, habitat loss, dam construction
Giant panda	M	M	L	L	M	L	L	Habitat availability/fragmentation
Chimpanzee	M	L	L	L	n.e.	M	L	Habitat loss, human conflicts, disease, food scarcity
Western gorilla	L	L	L	M	n.e.	M	L	Habitat loss and degradation, hunting and trade, disease
Eastern gorilla	M	L	L	L	n.e.	M	L	As western gorilla
Bonobo	M	M	L	L	n.e.	n.e.	L	Habitat loss, poaching
Bornean orangutan	L	L	L	L	n.e.	L	L	Habitat loss
Sumatran orangutan	L	M	L	L	n.e.	L	L	Habitat loss
Clouded leopard	M	M	L	L	n.e.	n.e.	L	No information
Bornean leopard	L	L	L	L	n.e.	n.e.	L	No information
Snow leopard	M	H	L	H	M	L	L	
Tiger	M	L	L	L	n.e.	L	L	Poaching, habitat loss and fragmentation, human conflicts
Polar bear					H	L	L	Habitat loss, human conflict, food scarcity
Marine species							L	
Loggerhead turtles					L	H		Long line fishing, egg predation
Hawksbill turtles					M	n.e.	M	Overexploitation, disease, incidental capture by fishermen, destruction of critical nesting habitat, human interactions.
Green turtle	L				H	L	L	Overharvesting and incidental capture, changes in beach sedimentology
Leatherback turtle	L				L	n.e.	L	Egg poaching, destruction or alteration of the nesting habitat, subsistence hunting, marine pollution, and the incidental capture
Olive ridley turtle	L				M	n.e.	M	Overexploitation, disease, incidental capture by fishers, and destruction of critical nesting habitat
Kemp’s turtle	L				n.e.	n.e.	L	Overexploitation, disease, incidental capture by fishers, destruction of critical nesting habitat
Blue whale	L				n.e.	M	L	No information found
North Atlantic right whale	L				M	n.e.	M	Food availability to allow recovery from past whaling
Bowhead whale	L				n.e.	n.e.	M	Low-level hunting by Inuit, habitat instability, predation
Fin whale	L				H (in Med.)	n.e.	M	Whaling, human disturbance, anthropogenic noise, pollution, collisions with ships
Gray whale	L				M	M	L	Human disturbances
Sei whale	L				n.e.	n.e.	M	Historically commercial whaling
							M	

Exposure for all marine species was low. There were some regions where the SST increases were relatively high (more than 2 °C), especially in the Mediterranean and temperate Pacific in the northern hemisphere. The distribution ranges of some turtle species include these regions, but as their ranges cover most of temperate oceans, the exposure was assessed as low.

The SRES A2 scenario projected slightly greater changes in both climate parameters (by 0.3 to 0.6 °C), such that just over half the species were in the medium exposure category. For precipitation, the percentage changes were also relatively small. If a SRES A2 scenario was realized then those in the medium category would be beyond the 2 °C threshold. Exposure alone is not necessarily sufficient to make a species vulnerable to climate change, as this will also depend on its sensitivity to these changes and its adaptive capability or for humans to put adequate adaptation measures in place.

### 3.2. Sensitivity

There is very little information on the sensitivity of most of the terrestrial species, except in the case of polar bears. Polar bears are assessed as highly sensitive, as they depend on sea ice as a platform for hunting seals, their primary prey, and reductions of sea ice extent and lengthening of the ice free season result in lower body conditions, reduced survival and population declines [30,31,32,33]. There is comparatively more evidence for marine species, particularly some turtles, and two species were assessed as highly sensitive: the green turtle and fin whale. There is evidence for green turtles of SST affecting different stages of their life cycle, while beach temperatures could be affecting breeding success [34,35]. The fin whale is thought to be sensitive only in the Mediterranean due to greater changes in SST and ocean circulation patterns here [36], which could affect its food supply.

Those in the medium category include, African elephants due to evidence of their sensitivity to drought, giant pandas for their specialization in food sources, hawksbill and Olive ridley turtles and gray whales. For hawksbill turtles in Antigua, air temperatures are already above the pivotal temperature for part of the nesting season [37] and elsewhere they are being affected by sea level rise [38,39] and storms [40], both of which are projected to increase. Olive ridley turtles in the central Northern Pacific Ocean were found to primarily occupy a 5 °C range, (23–28 °C) during all seasons, but within that they appeared to be found at SST centered on 24 and 27 °C [41], thus as SST increase they could experience temperatures outside their preferred range and, if they were not able to adapt, either physiologically or by migrating to cooler areas, they would be vulnerable. 

### 3.3. Adaptive Capability

The adaptive capability of species was also dependent on evidence availability: again, more evidence was available for marine species. Green turtles were classified as low adaptive capability, as although they are exhibiting small shifts in breeding, it is thought that the pivotal temperature, leading to a sex ratio imbalance will be more critical [42]. This could be important for many other marine turtles, and although no explicit supporting evidence was found, it was implied in several papers. Recent research on green turtles nesting behavior, however, has suggested that their adaptive capability may have been underestimated [43]. In addition, sea level rise could lead to the loss of beach habitat (affecting adaptation opportunity), especially where movement inland is not possible because of human developments [44]. For gray whales, a study of their movements in Magdalena Bay, Mexico showed that during El Niño years, when SST can be 4–5 °C above average, the number of whale sightings decreased [45]. This suggests sensitivity, possibly combined with adaptive capability if they had migrated to cooler waters, although this was not recorded. 

Loggerhead turtles were assessed as high adaptive capability, as they have a number of adaptation options [46] and there is some evidence of them already adapting. For example, the timing of nesting is already changing in response to increases in SST [40,47]. Other species also showing evidence of adaptation to current climate changes and which are categorized as having medium adaptive capability include:

- African and Asian elephants which may migrate (temporarily) to different areas in periods of drought to utilize a wider range of food [48,49];
- Chimpanzees, which can alter the amount of time spent on the ground in response to temperature [50];
- Gorillas, which have a certain flexibility in their diet to cope with lean times [51,52];
- Blue whales, which are thought to be able to track changes in SST frontal zones, which are important feeding areas [53];
- Gray whales are staying longer in the Arctic in response to increased SSTs [54], leading to later southward migrations [55].

### 3.4. Adaptation Opportunity

For some species, adaptation may be more a function of low adaptation opportunity, as other pressures, such as habitat loss and fragmentation and various human interactions are more immediate and prevent their movement in response to changing climatic conditions. Asian rhinoceros, orang-utans and giant pandas [56], for example, have small populations often in isolated pockets in fragmented habitats. Similarly, tigers are thought to have a high adaptive capability, as they can live under a wide range of climatic and habitat conditions, and can utilize various types of prey [57], but habitat loss and fragmentation, alongside hunting and poaching are seriously reducing the opportunity for expression of their adaptive capability [58,59]. This illustrates the importance of considering both adaptive capability and opportunity to get a fuller picture of adaptive capacity.

For the terrestrial mammals, all were considered to have a low adaptation opportunity as they face a number of pressures. The factors mentioned most frequently as affecting this were poaching and hunting, habitat loss and/or degradation and/or fragmentation. The former constituted one of the major causes of the population declines of most species in the past, and still continues to be a great threat to some species, such as elephants, rhinoceros, and tigers, while this threat has declined for species, such as giant pandas and polar bears. Habitat changes are affecting a number of species and they are probably the most important threat to the selected species, at least in the short-term. They not only affect population numbers and the area they can inhabit, but they also impact on food availability and may, as in the case of primates, elephants and tigers, lead to human conflicts as agricultural areas are raided or livestock attacked. Such human conflicts may increase due to climate change; for example, agricultural and grazing areas may expand to higher altitudes as they become climatically suitable, while alpine species, such as snow leopard, increasingly raid crops and livestock in response to loss of habitat and natural prey [60]. Thus, the success of a species exploitation of their adaptation opportunity may be affected by social responses, including how humans value and utilize wildlife, which can themselves be affected by pressures from climate change. 

For marine species, adaptation opportunity seemed to revolve around pressures that decreased population numbers and limited species responses. For example, for marine turtles, over-exploitation and bycatch were the most frequent factors identified as causing decline in populations; while measures are being implemented in many areas to reduce these, recovery can be slow in the face of other pressures. Historically, for whales, commercial whaling was the major cause of population declines, but few current factors were reported in the literature. A global assessment of human impacts on the marine environment, however, suggested that factors related to climate change (ocean acidification, ultra violet light levels and SST) are affecting the greatest area of the ocean, followed by fishing [61].

## 4. Discussion

There are a number of limitations, stemming from both the availability of evidence in the literature and the analysis of exposure. The availability of relevant research or monitoring of species or population responses to current climate affects the ability to categorize robustly a species' sensitivity and adaptive capability and, to a lesser extent, opportunity. This may lead to species being classed as medium or high sensitivity or adaptive capability as a result of more information. The results, therefore, are indicative, not definitive and should be updated as new research becomes available. It highlights the need for specific research on understanding of climate change effects on existing drivers or threats to such species, to inform efforts to enhance species adaptation opportunity and avoid maladaptation. 

For exposure the difference between climate projections between GCMs and emissions scenarios is relatively small for temperature extremes, but large for precipitation extremes [17] leading to higher uncertainty in species sensitivity assessment. In addition, given the projected changes varied across a species range not all populations are equally exposed. The calculation of exposure based on the mean changes across the range can undermine the population level impacts, especially for threatened terrestrial species with small isolated populations. In addition, more indirect climate-related factors, such as sea level rise or changed disturbance regimes (e.g., fire), can have important consequences for species and their vulnerability, as well as for human systems which can lead to increased human-wildlife conflicts, e.g., through poaching.

The adaptive capability primarily depends on species inherent biological characteristics (or traits) as used by the IUCN [11]. For many species the existence and nature of the limits of adaptive capability are unknown, and the adaptive capabilities used here do not take into account the possibilities of their capability being exceeded by the future magnitude or rates of climate change or the magnitude or frequency of extreme events, thus, the conclusions may be optimistic. While humans can, as part of planned adaptation, alter these through genetic breeding, artificially increasing population sizes and translocation [62,63], humans primarily affect adaptation opportunity through being responsible for other stresses which decrease populations and their viability, as well as destroying and fragmenting habitat and creating other barriers to species movement. These pressures maybe more significant in the short term or their interaction with climate change can additionally affect the species vulnerability [64,65] and can themselves be driven by climate change.

Vulnerability assessment frameworks using exposure, sensitivity and adaptive capacity have been applied to many species and ecosystems [11,12,66,67,68,69,70,71]. A comparison of some of these found that the measures used to estimate these three components differ (Table 7). Generally, they considered exposure as the rate and magnitude of climate change in species ranges or habitats [6,11,70], either expressed by the extent of species ranges under climate changes [10,68], extent of the overlaps in species ranges between current and future climates [9], or frequency of the habitat being affected [67]. However, the distinctions between sensitivity and adaptive capacity are more ambiguous. For example, some defined species sensitivity as the changes in the probability of occurrences within species ranges [10,65], while others [69] defined sensitivity as the level that each habitat will be impacted, indicated by temperature increases or loss of habitat. Some authors considered sensitivity as intrinsic traits of species, such as physiological tolerance [68], habitat preference or dependency [6,9], or phenotypic variation [65,66,67,68,69,70], although physical tolerance [67,71] and phenotypic variation [6] could also be included as adaptive capacity. Dispersal ability or migration is commonly considered as a measure of adaptive capacity [6,9,10,68,69,70,71], as are lack of barriers [69,70] or landscape permeability [12,70]. The variations in measures used are partly due to differences in how the components are defined and the traits of target species, but also the purpose of the assessment and data availability. Any climate change vulnerability assessment for conservation planning should include exposure, sensitivity and, as shown, both adaptive capability and adaptation opportunity, and within this as many of their components as are relevant and possible given the paucity of ecological data for many species. 

**Table 7:** A comparison of the components used in a selection of conservation-related vulnerability assessments and how they were classified. E = Exposure; S = Sensitivity; AC = Adaptive capacity.

	Williams [68]	NatureServe [69]	Galbraith and Price in NWF [12]	Lawler *et al*. in NWF [70]	Gardali [71]	Foden [11]	Berry *et al*.(this paper)	Description(from Table 1)
**Exposure—Extrinsic effects resulting from climate change**	Regional climate change and local microhabitat buffering	Magnitude of predicted temperature and moisture change across the species range (E); Sea level rise (IE)	Character, magnitude and rate of change the physical system or species is likely to experience, as a results of climate, disturbance regimes, shifts in vegetation type and salinity changes, drought, fire, CO_2_ *etc*.	Historic observed changes in climate; future modeled projections; baseline climate; drought; hydrology; fire regimes; CO2 concentrations; vegetation; salinity; pH; storms	Extrinsic factors (e.g., increasing temperatures or habitat loss) resulting from climate change; changes in extreme weather	Climate change	Magnitude of predicted temperature changes across the range for all species and rainfall changes for terrestrial mammals only	Climate change in the species range (incl. extremes, sea level rise)
			Likely extent of habitat loss due to climate change; habitat ability of to shift at the same rate as species	Species distribution changes	Changes in habitat suitability			
**Sensitivity—Species' traits (intrinsic factor) related to susceptibility to climate change**	Restricted ranges		Degree of habitat specialization	Degree of habitat specialization	Habitat specialization	High degree of habitat specialization	Associated with restricted habitats	Habitat dependence/preference
		Dependence on ice, ice-edge, or snow-cover habitats		Dependence on sensitive habitats		Restricted to habitats susceptible to climate change; Narrow altitudinal range and a high elevation	Associated with habitats sensitive to climate change	
		Restriction to uncommon geological features or derivatives				Dependence on a particular microhabitat		
	Physiological tolerance limits	Predicted sensitivity to temperature and moisture changes	Physiological vulnerability to temperature change/precipitation change	Physiological factors	Physiological tolerance	Global temperature tolerances likely to be exceeded	Physiological tolerance limits	Physiological sensitivity to climate changes and extreme events; dependence on environmental triggers
	Lack of ability to survive and recover from disturbance	Dependence on a specific disturbance regime likely to be impacted by climate change (S)	Vulnerability to climate change-induced extreme weather events. Sensitivity to wind, fire and/or hydrological regimes			Vulnerable to physical damage from storms and cyclones	Sensitive to extremes e.g., drought	
			Dependence on temporal inter-relationships			Environmental trigger/cue disruption observed or likely	Dependence on environmental triggers	
		Phenological response to seasonal changes		Phenology changes				Phenology changes
		Reliance on interspecific interactions	Dependence on other species	Degree of specialization in food sources		Dependent on very few prey or host species	Specialist feeder	Interspecific interaction
				Ecological linkages		Dependent on an interspecific interaction that is likely to be impacted by climate change		
						Susceptible to enigmatic decline		
	Life history traits			Reproductive strategy			Life history traits	Life history traits
	Population dynamics			Population growth rates				Population dynamics
**Adaptive capacity—Intrinsic factor related to species ability to cope with climate change**	Dispersal ability (S)	Poor dispersal ability (S)	Dispersive capability	Dispersal abilities (Sp. AC)	Dispersal ability (S)	Low maximum dispersal distances	Dispersal ability	Species’ ability to disperse or colonize
	Genetic diversity	Measured genetic variation (S/AC) or occurrence of bottlenecks in recent evolutionary history (S)						
	Ecological plasticity		Ecological plasticity (AC)	Plasticity (Species AC)			Ecological plasticity	Ecological plasticity
	Evolutionary potential		Evolutionary potential (AC)	Evolutionary potential (Species AC)			Evolutionary potential	Evolutionary potential
			Functional redundancy (AC)	Functional redundancy (Ecological AC)				
**Adaptive opportunity—Extrinsic factors limiting species' ability to cope with climate change**	Biogeographic connectivity	Distribution relative to natural and anthropogenic barriers, impact of land use changes from human responses to climate change (Indirect E)			Geographic barriers (S)		Geographic barriers	Limited opportunity for species to disperse or colonize
			Landscape permeability	Landscape permeability (Habitat AC)			Landscape permeability	
			Habitat availability within new range of species		Changes in food availability (E)		Habitat or food availability	Gain/loss of potential habitats

Assessment of adaptive capacity is particularly important for species sensitive to climate change, as it enables better identification of the source of any limitation in capacity, that is whether it is inherent to the species biology or is a function of its environment. It can also provide direction for conservation, while adaptation opportunity indicates where conservation action is most needed.

The distinction leads to a fourfold classification of species, which could inform conservation action (Table 8), although in reality the classes will not be so clear cut, especially for species with large ranges:

i) Climate resilient—climate change is currently not a major threat, because species can adapt, although, as with all species, monitoring is needed to ensure that both the adaptation capability and opportunity are maintained. Possible climate resilient species include loggerhead turtles, which while they have high adaptive capability [40], their opportunity is unknown, and only assumed to be at least medium.
ii) Opportunity restricted—this category primarily applies to terrestrial mammals and includes Asian elephants, African rhinos, tigers and gorillas. Asian elephant populations, for example, are affected by shifting cultivation, encroachment, poaching, mining, forest fires, scarcity of water during dry season, increase in human populations leading to human-elephant conflict and mortality due to diseases, electrocution [72]. In addition, their current populations are highly isolated, due to the loss and fragmentation of habitat [73].

Increasing opportunity could include enhancing existing conservation measures, whilst planning and preparing for longer term climate change, alongside reducing non-climate pressures. For African elephants and rhinoceros, water provision, especially in the dry season and drought years, has been cited as the main direct management intervention relevant for climate change and available to managers of arid or semi-arid conservation areas. While this has been used in Africa in various parks, there is debate about its effectiveness in achieving animal dispersal and protecting vegetation in drought periods [62]. The use of fire, culling and translocation have been suggested as the other main options available to managers of big game [63]. Translocation as a means of metapopulation management is a further possible option, for example, in Pilanesberg when black rhinoceros densities become too high, as this would also help maintain genetic diversity [74]. This could be an option for many other species, providing that there are suitable areas available, but it is costly and involves a good knowledge of the ecology of the species.

**Table 8:** Classification of species vulnerability and potential conservation responses.

Adaptation opportunity	Adaptive capability	
	High	Low
High	(i) climate resilient—monitor the species; monitor habitat condition and availability of habitat for migration	(iii) capability restricted—modify microclimate; minimise habitat and other ecological changes; ensure availability of food/habitat for specialists; increase population numbers
Low	(ii) opportunity restricted—increase habitat area; increase connectivity; remove barriers to movement; reduce current pressures; ensure availability of food	(iv) climate threatened—consider all actions for opportunity and capability restricted species; translocation, *ex situ* conservation

In the marine environment, a range of possible climate change adaptation opportunity measures have been suggested [66,75]. Given the problem of pivotal temperatures and their effect on sex ratios, focusing conservation on beaches, which produces a higher ratio of males, would be effective. In addition, sand temperatures could be modified by artificial shading, increasing vegetation cover through re-forestation, sprinkling cool water to try and obtain favorable pivotal temperatures [76]. Measurements at Junquillal Beach, Costa Rica indicated that the coastal vegetation strip can reduce incubation temperature by 2–3 °C along the higher elevation stretch of the beach [77]. Alternatively, nests could be re-located to more suitable incubating environments. These management options have been suggested as being more practical at smaller rookeries, due to labor requirements and financial costs [78]. For leatherback turtles, the relocation of eggs from areas that are in danger of erosion, poaching, or predation is a widespread conservation management practice that has been shown to be effective [79,80,81], although there is some evidence that *in situ* nests had greater hatching success [82].

iii) Capability restricted—it is harder to enhance adaptive capability directly, except through breeding. Conservation actions could focus on minimizing changes (and other pressures) so as to maintain population numbers and reduce exposure to climate change. Orang-utans possibly come into this category, although habitat loss is the only mentioned factor affecting opportunity, but given current rates of deforestation it is likely that they could soon be category iv. Attempts to reduce exposure in order to decrease the need for adaptive capability have been undertaken through the provision of watering holes for African elephants and modifying the sand temperatures (see above).
iv) Climate threatened—this includes a number of terrestrial species, such as the Sumatran rhino, which experience multiple pressures restricting them to small isolated populations. These are the most climate change threatened species and all adaptive capability and opportunity actions should be considered. Actions could include identifying those pressures that are having the greatest effect or can be most easily addressed.

The separation of adaptive capacity into adaptation capability and opportunity indicates that most of the species have an inherent level of ability to respond to climate change, which is not surprising given that climate is always changing and thus adaptation has been a feature of their existence. Human pressures, however, in many cases are reducing the possibility of species responding, thus diminishing their adaptation opportunity. From a conservation perspective, addressing these other immediate pressures on species could be an effective action in the short-term.

There are a number of conservation management actions designed to tackle these and enhance adaptation, but currently there is comparatively little evidence of their effectiveness. In addition, some may represent maladaptation in that they decrease adaptation opportunity. For example, for African elephants, research has shown that the provision and management of artificial waterholes can affect the distribution of large herbivores, especially in drought years, by hindering species migration and that this migration response also can be hindered by fences round reserves [47,59,83,84]. The framework, therefore, combined with the classification of adaptation components (Table 8), can provide clear guidelines on which species are vulnerable and the type of actions that are most needed in order to facilitate the species adaptation to climate change.

## 5. Conclusions

This study has shown the difficulty of providing a consistent, comprehensive assessment of the climate change vulnerability of a selection of threatened species, due to the lack of information for many of them. Nevertheless, based on available evidence and subjective categorization, many possible sources of vulnerability could be identified. The separation of adaptive capacity into adaptation capability and adaptation opportunity enhanced the usefulness of vulnerability assessments for conservation through enabling the identification of whether the species was inherently limited in its ability to cope with climate change or whether environmental and human factors constrained its adaptive capability, or both. It also showed that species adaptation opportunity was particularly important for the terrestrial mammals due to the existence of multiple pressures. This framework, therefore, provides clear guidance for identifying appropriate conservation adaptation actions to enhance species resilience to climate change, thus addressing one of the pressing concerns of the 21st century.
